# Advancing β-adrenoreceptor agonism for recovery after volumetric muscle loss through regenerative rehabilitation and biomaterial delivery approaches

**DOI:** 10.1093/rb/rbaf015

**Published:** 2025-03-19

**Authors:** Jennifer McFaline-Figueroa, Christiana J Raymond-Pope, Joseph J Pearson, Albino G Schifino, Junwon Heo, Thomas J Lillquist, Emma E Pritchard, Elizabeth A Winders, Edward T Hunda, Johnna S Temenoff, Sarah M Greising, Jarrod A Call

**Affiliations:** Department of Physiology and Pharmacology, University of Georgia, Athens, GA 30602, USA; School of Kinesiology, University of Minnesota, Minneapolis, MN 55455, USA; W.H. Coulter Department of Biomedical Engineering, Georgia Institute of Technology and Emory University, Atlanta, GA 30032, USA; Department of Physiology and Pharmacology, University of Georgia, Athens, GA 30602, USA; Department of Physiology and Pharmacology, University of Georgia, Athens, GA 30602, USA; School of Kinesiology, University of Minnesota, Minneapolis, MN 55455, USA; School of Kinesiology, University of Minnesota, Minneapolis, MN 55455, USA; Department of Physiology and Pharmacology, University of Georgia, Athens, GA 30602, USA; Department of Physiology and Pharmacology, University of Georgia, Athens, GA 30602, USA; W.H. Coulter Department of Biomedical Engineering, Georgia Institute of Technology and Emory University, Atlanta, GA 30032, USA; Petit Institute for Bioengineering and Bioscience, Georgia Institute of Technology, Atlanta, GA 30332, USA; School of Kinesiology, University of Minnesota, Minneapolis, MN 55455, USA; Department of Physiology and Pharmacology, University of Georgia, Athens, GA 30602, USA; Regenerative Bioscience Center, University of Georgia, Athens, GA 30602, USA

**Keywords:** muscle torque, mitochondrial bioenergetics, muscle injury, muscle recovery, formoterol

## Abstract

Volumetric muscle loss (VML) injury results in the unrecoverable loss of muscle mass and contractility. Oral delivery of formoterol, a β_2_-adrenergic receptor agonist, produces a modest recovery of muscle mass and contractility in VML-injured mice. The objective of this study was to determine if a regenerative rehabilitation paradigm or a regenerative medicine paradigm could enhance the recovery of VML-injured muscle. Regenerative rehabilitation involved oral formoterol delivery combined with voluntary wheel running. Regenerative medicine involved direct delivery of formoterol to VML-injured muscle using a non-biodegradable poly(ethylene glycol) biomaterial. To determine if the regenerative rehabilitation or regenerative medicine approaches were effective at 8 weeks post-injury, muscle mass, contractile function, metabolic function, and histological evaluations were used. One model of regenerative rehabilitation, in which rehabilitation was delayed until 1 month post-injury, resulted in greater muscle mass, muscle contractility, and permeabilized muscle fiber mitochondrial respiration compared to untreated VML-injured mice. Histologically, these mice had evidence of greater total muscle fiber number and oxidative fibers; however, they also had a greater percentage of densely packed collagen. The regenerative medicine model produced greater permeabilized muscle fiber mitochondrial respiration compared to untreated VML-injured mice; however, the non-biodegradable biomaterial was associated with fewer total muscle fibers and lower muscle quality (i.e. lower muscle mass-normalized contractility). The conclusions reached from this study are: (i) regenerative rehabilitation and regenerative medicine strategies utilizing formoterol require further optimization but showed promising outcomes; and (ii) in general, β-adrenergic receptor agonism continues to be a physiologically supportive intervention to improve muscle contractile and metabolic function after VML injury.

## Graphical abstract

**Figure:**
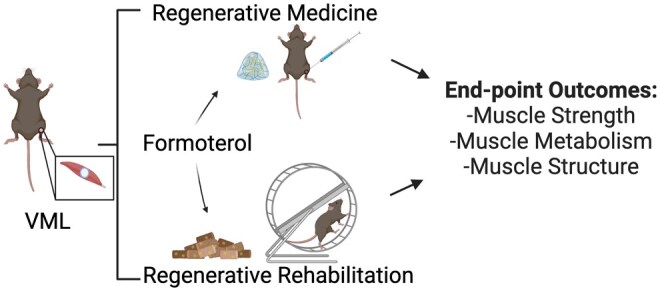

## Introduction

Sympathomimetic drugs have long been used to treat cardiac, pulmonary, neurologic, and endocrine diseases, among others, through the activation of α- and β-adrenergic receptors [1]. Of these drugs, β_2_-adrenergic receptor agonists have shown therapeutic potential in conditions of muscle wasting and muscle loss through the activation of G-protein-coupled receptors leading to hypertrophy, increased strength, and enhanced repair [2, 3]. For example, oral salbutamol, a long-lasting β_2_-agonist bronchodilator found in fast-acting inhalers, has been shown to alleviate muscle atrophy in a murine model of diabetes by reducing oxidative stress and inflammation [4, 5], help maintain muscle mass and improve muscle cross-sectional area in denervated soleus muscles [6], and improve strength and protein turnover in human skeletal muscle [7–9]. Formoterol, another drug in this same class, has been used to improve oxidative capacity of skeletal muscle [10, 11], muscle force production [12], mitochondrial biogenesis and function [13–15], and to promote regeneration and hypertrophy [11, 16, 17] when delivered orally across several disease and injury models.

Volumetric muscle loss (VML) injury is characterized by unrecoverable loss of muscle mass and function due to trauma or surgery [18]. These injuries result in the marked decline of muscle strength and patient mobility [18–20], as well as the increase of fibrotic tissue deposition [19, 21–24]. VML injury is also associated with a decline of skeletal muscle and whole-body metabolism, such as depressed mitochondrial respiration in permeabilized muscle fibers [11, 23, 25], decreased mitochondrial enzyme activity [11, 26, 27], less activation of mitochondrial biogenesis markers [23, 27], and changes in whole-body diurnal metabolism, resulting in lower oxidation of carbohydrates [24, 28]. Traditional rehabilitation techniques alone have not been successful in significantly rescuing contractile and metabolic function in these injuries [23, 25], prompting investigations into alternative strategies.

β-Agonism represents one alternative strategy to mitigate the comorbidities of VML. Previously, the use of formoterol as a therapeutic following VML injury resulted in the improvement of contractile and metabolic function [11] and changes in whole-body metabolism, such as increased flexibility in energetic substrate utilization [28], compared to similarly injured, untreated cohorts. When combined with leucine, formoterol treatment attenuates the loss of total muscle fibers and partially rescues contractile function [29]. While these results show the promise of formoterol as a potential treatment for VML injury, they did not completely restore muscle mass and function in VML-injured limbs.

The primary objective of this study was to determine if the therapeutic effect of formoterol in the context of VML injury could be enhanced by either: (i) combining formoterol administration with rehabilitation (i.e. regenerative rehabilitation), or (ii) direct, sustained delivery of formoterol to skeletal muscle via a biomaterial. Regenerative rehabilitation is the practice of combining regenerative therapies (e.g. pharmaceuticals, growth factors, scaffolds, stem cells, etc.) with means of rehabilitation to determine whether the sum of these approaches is greater than each isolated element. In this case, it represents a holistic, whole-body approach to VML injury therapy consisting of formoterol and voluntary wheel running. Conversely, the use of a biomaterial was used to directly deliver formoterol to the area of injury to determine the extent to which localized treatment of the injury results in greater functional rescue.

## Materials and methods

### Ethical approval of animal use

Male C57BL/6J (The Jackson Laboratory; stock: 000664) mice were housed at 20–23°C on a 12:12-hour light-dark cycle, with food and water provided *ad libitum*. All mice were between age 10 and 11 weeks (pre-surgical mass: 28.16 ± 1.56 g) when enrolled and randomized into experimental cohorts. The Institutional Animal Care and Use Committee at the University of Georgia and the University of Minnesota approved all procedures.

### Experimental design

**Figure 1.**
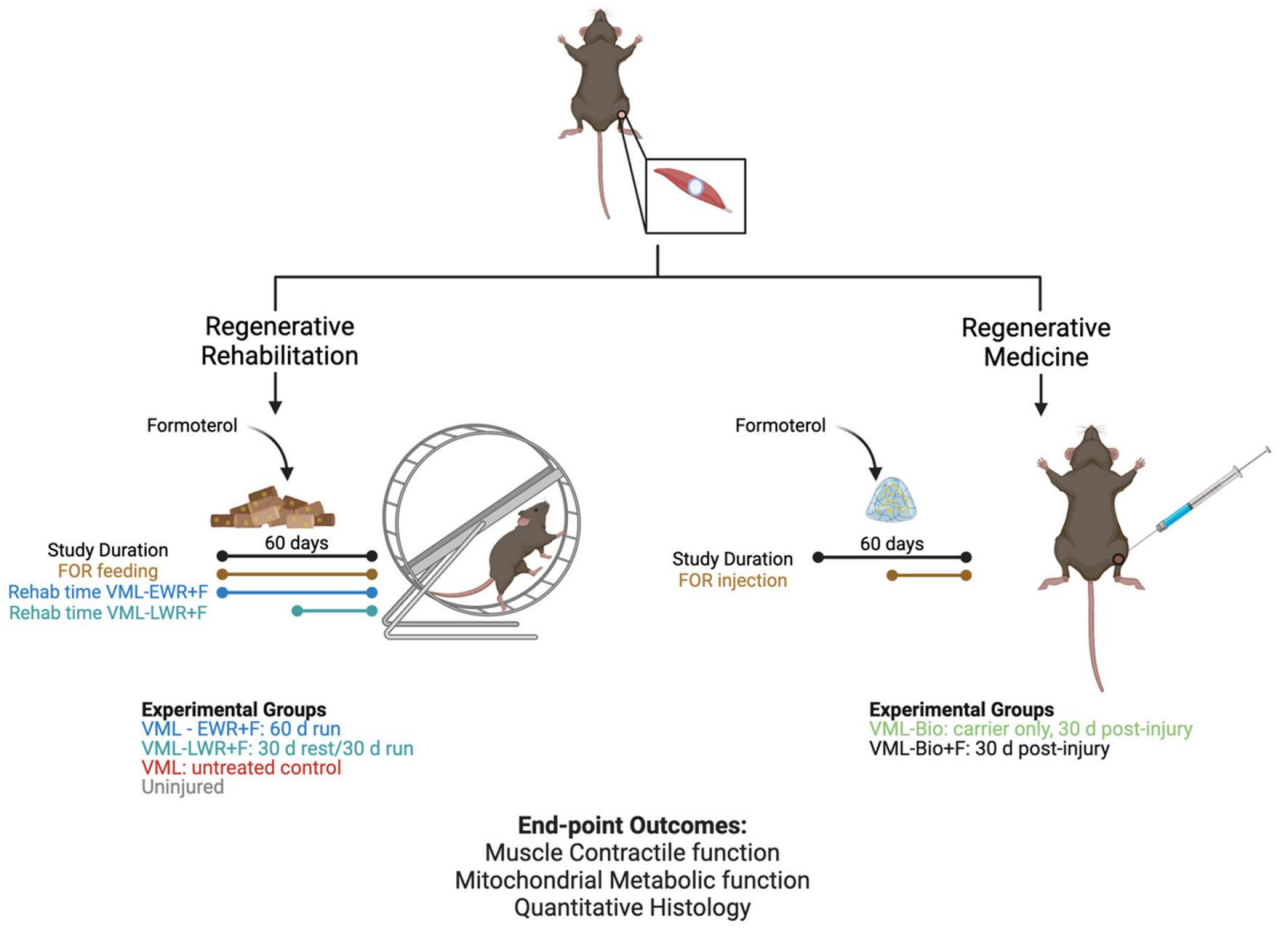
Study design graphic. VML-injured animals were randomized into regenerative rehabilitation and regenerative medicine experimental study wings. Regenerative rehabilitation involved 60 days of oral formoterol with either 60 days of voluntary wheel running (early rehab) or 30 days of voluntary wheel running (late rehab). Regenerative medicine involved delivery of formoterol-laden poly(ethylene glycol) non-biodegradable biomaterial beneath the muscle fascia at 30 days post-injury. Muscle contractile and metabolic function was assessed at 60 days post-injury, immediately prior to muscle isolation for quantitative histology.

Two control groups and four experimental groups were included in the primary study design (Figure 1). Injury naive mice were used as uninjured controls (*n* = 8) and VML-injured mice without further manipulation were used as VML-untreated controls (*n* = 14). The four experimental groups included two groups receiving regenerative rehabilitation and two groups receiving biomaterial, all after a VML injury. The regenerative rehabilitation experimental groups were provided oral formoterol in mixed chow (TestDiet, Richmond, IA; 0.3 mg/kg/day) as previously described [11, 28] starting at 3 days post-injury, and then given access to voluntary running wheels at either 7 days post-injury for 60 days (early wheel running, VML-EWR+F, *n* = 8) or at 1 month post-injury for 30 days (late-wheel running, VML-LWR+F, *n* = 8). The rationale for the different wheel access times was to determine if initiating rehabilitation early vs. late influenced muscle function in the context of formoterol.

The biomaterial experimental groups were injected (intramuscular) with blank biomaterial (VML-Bio, *n* = 8) or formoterol-laden biomaterial (VML-Bio+F, *n* = 9) at 1 month post-VML injury. The rationale for the biomaterial timeline was to allow the remaining muscle pathophysiology to stabilize before introducing a foreign agent (i.e. biomaterial) [30]. The study endpoint for all animals was 2 months post-VML injury and this timeline spanned age 12–20 weeks, in agreement with previous VML and formoterol studies [11, 28].

After completion of the study design above, an additional cohort of mice was added for histological assessment of the VML-injured gastrocnemius muscle. This cohort of mice included uninjured controls (*n* = 3), untreated VML (*n* = 3), VML-injured with oral formoterol-only (*n* = 3), VML-LWR with oral formoterol (VML-LWR+F; *n* = 3), and VML-Bio+F (*n* = 3). The experimental timeline was the same as above, with VML injury occurring at age 12 weeks and the study endpoint at age 20 weeks. All cohorts of mice were euthanized by CO_2_ inhalation followed by cervical dislocation. Following euthanasia, the gastrocnemius muscle was isolated for physiological, biochemical, or histological assessment.

### Surgical creation of VML injury

A unilateral VML injury to the posterior compartment of the left hindlimb was created in anesthetized mice (isoflurane 1.5–2.0%) using a 4-mm biopsy punch as previously described [23, 25]. Across mice, the average tissue removed was a 28.4 ± 1.6 mg portion. After closing the wound with 5-0 vicryl sutures, analgesic was provided for 72 hours post-procedure.

### Voluntary wheel running

Individually housed mice were given free access to a running wheel (Columbus Instruments, Columbus, OH) [23, 31]. Daily running totals were calculated from wheel revolutions collected at 5 min intervals and are presented as a daily distance averaged per week.

### Biomaterial (PEG-DA) synthesis

The 3.4 kDA poly(ethylene glycol) (PEG) was functionalized, as previously described [32]. Briefly, 3.4 kDa PEG was dissolved in distilled methylene chloride (DCM) in a three-neck round-bottom flask and stirred under nitrogen flow. Triethylamine in DCM was added to the PEG at a 1:1 ratio. Subsequently, a 100% molar excess of acryloyl chloride (AcCl) in 15–20 ml of DCM was added dropwise and then closed and kept under nitrogen overnight. To remove the trimethylamine hydrochloride by-product, potassium carbonate (K_2_CO_3_) was added to the solution and this allowed for separation into organic and aqueous phases. After separation of the organic phase, the PEG-DA was precipitated using cold diethyl ether and dried overnight at room temperature. A sample of dried PEG-DA was diluted in deuterated water (D_2_O) and analyzed using proton nuclear magnetic resonance for the presence of acrylate peaks around 5.8–6.4 ppm. The finished product was stored at –20°C until use.

### Biomaterial fabrication

PEG-DA hydrogel fragments were fabricated as per Pearson *et al*. [33]. PEG-DA was combined at a 10 wt% total solution ratio with sterile phosphate buffered saline (PBS), thermal initiators ammonium persulfate (APS, 0.018M) and N, N, N′,N′-tetramethylethane-1,2-diamine (TEMED, 0.018M) and bovine serum albumin (BSA) equivalent to 20 wt% of the solution. The solution was pulled up into a 1-ml syringe and allowed to gel at 37°C. Following crosslinking, the mixture was pushed through a 40-µm filter into a microcentrifuge with sterile PBS. This process was repeated three more times before being passively filtered to remove gel pieces smaller than 40 µm. Gel fragments were imaged at ×20 magnification and sized using FIJI.

### Formoterol-loading and *in vitro* release

Approximately 500 µl of filtered and sized gel fragments were centrifuged, and all excess PBS was aspirated. Formoterol (5.7 mg/ml) was dissolved in sterile PBS containing 5% dimethyl sulfoxide (DMSO), to aid dissolution. Lost volume was replaced with 5% DMSO PBS with or without formoterol and vortexed to ensure thorough mixing. Fragments were loaded overnight at 4°C. After loading, each tube was filled with 500 µl of PBS and incubated at 37°C for the release study. PBS was exchanged at appropriate timepoints and stored at −20°C for subsequent analysis. *In vitro* release of formoterol was analyzed using methyl orange, as described previously [34]. Briefly, release samples were adjusted to pH 4.0 and mixed with equal parts methanol and methyl orange dye. Formoterol was extracted with chloroform, vortexed for 1 min, and 3000 rpm for 2 min.

### Biomaterial delivery

VML-injured mice were allowed to recover without intervention for 1 month before being randomly divided into the blank or formoterol-laden biomaterial experimental groups (i.e. VML-Bio and VML-Bio-F, respectively). Mice were anesthetized under isoflurane (1.5–2%), hair was removed, and skin was aseptically prepared for biomaterial injection. 100 µl of biomaterial was injected beneath the muscle fascia of the affected limb with a 25 G needle.

### *In vivo* muscle strength

Left hindlimb plantar flexor muscles (soleus/gastrocnemius/plantaris muscles) were assessed for peak-isometric torque *in vivo* as previously described [35, 36]. Using a servomotor (Model 300C-LR; Aurora Scientific, Aurora, Ontario, Canada) and percutaneously placed platinum-iridium needle electrodes (Model E2-12; Grass Technologies, West Warwick, RI), peak-isometric torque, defined as he greatest torque measured during a 250-ms stimulation using 1-ms square-wave pulses at a frequency ranging from 150 to 200 Hz, was measured. *In vivo* passive stiffness was assessed as previously described [37, 38].

### Exhaustive treadmill capacity test

Uninjured mice, untreated VML mice, and mice enrolled in the regenerative rehabilitation study performed an exhaustive treadmill capacity test 1 week prior to euthanasia to confirm whole-body endurance adaptation in the regenerative rehabilitation cohorts. The treadmill protocol was initiated on the fourth day, after three consecutive days of treadmill familiarization (Columbus Instruments). The protocol consisted of two stages, the first involving 7 min at 7.5 m/min and the second involving 7 min at 10 m/min. The protocol proceeded to increase speed by 2.5 m/min at 10 min increments until 27.5 m/min maximal speed was reached [39]. The protocol ended when mice did not respond to five consecutive taps with brushes. The final distance was recorded for the mice.

Percentage grade remained constant throughout the test at 5%. Brushes at the end of the treadmill lane encouraged mice to keep running throughout the test. The fatigue test was terminated when mice no longer responded to five consecutive, forceful taps with the brushes. Treadmill distance and time were recorded for all mice.

### Permeabilized muscle fiber mitochondrial respiration

Mitochondrial respiration was performed on bundles of permeabilized fibers from the gastrocnemius muscle using a high-resolution Oroboros Oxygraph-2K instrument (Oroboros Instruments, Innsbruck, Austria) as previously described [40, 41] with slight modifications. The experiments were conducted at 30°C in buffer Z (105 mM K-MES, 30 mM KCl, 1 mM EGTA, 10 mM KH_2_PO_4_, 5 mM MgCl, 0.5 mg/mL BSA; pH 7.1) containing ATP (5 mM), creatine (5 mM), phosphocreatine (PCr, 1 mM) and creatine kinase (20 U/ml). To test mitochondrial respiration (*J*O_2_) and electron conductance via the electron transport system (ETS) under a physiologically relevant environment, we employed a creatine kinase (CK) clamp system where this assay depends on the enzymatic reaction of CK and phosphocreatine (PCr) to control the levels of cellular energy demand (Gibbs-free energy; ΔG_ATP_) [41]. For the experiment, permeabilized fiber bundles from gastrocnemius muscle energized using 10 mM glutamate, 5 mM malate and 10 mM succinate to mimic near-exercise conditions (maximal *J*O_2_), followed by sequential PCr titrations (1, 2, 4, 7, 16, and 31 mM) to reduce ΔG_ATP_ to resting conditions. The measured rates were normalized to tissue wet weight.

### Mitochondrial membrane potential

Mitochondrial membrane potential (mtMP) was fluorometrically performed at 30°C in 0.2 ml of buffer Z supplemented with ATP (5 mM), CK (200 U/ml), and creatine (20 mM) using a Horiba Spectrofluorometer (FluoroMax Plus-C; Horiba Instruments Inc., Atlanta, GE, USA) as previously described [26]. The mtMP was determined via tetramethylrhodamine methyl ester with excitation/emission (572/590 nm)/(551/590 nm). Permeabilized muscle fibers were weighed (1–1.5 mg) and placed into cuvettes containing the buffer, followed by adding substrates 10 mM glutamate, 1 mM malate and 10 mM succinate to energize mitochondria, and then sequential PCr titrations were made to final concentrations of 1, 2, 4, 7, 16 and 31 mM, which is parallel with the mitochondrial respiration assays.

### Assessment of mitochondrial respiratory complex activity

Mitochondrial enzyme activities, citrate synthase (CS), and complex I/II enzyme activities were determined using a spectrophotometer (Molecular Devices, San Jose, CA, USA) as previously described [11, 40]. For these assays, muscle homogenate was further diluted with 10 mM phosphate buffer. Mitochondrial content was tested by CS activity as previously described [41]. Complex I activity was measured in 50 mM potassium phosphate buffer, 3 mg/mL BSA, 0.4 µM antimycin A, 240 µM potassium cyanide, 50 µM decyl-ubiquinone, and 80 µM 2, 6- dichlorophenolindophenol (DCPIP). The oxidation of nicotinamide adenine dinucleotide (NADH) was measured via the reduction of DCPIP at 600 nm. Complex II activity was assessed in a buffer containing 10 mM KH_2_PO_4_, 2 mM ethylenediaminetetraacetic acid (EDTA), 1 mg/ml BSA at pH 7.8 and added with 200 µM ATP, 10 mM succinate, and 80 µM DCPIP. Following incubating the buffer for 10 minutes at 37°C, the assay was initiated by adding oxidized 80 µM decyl-ubiquinone, and reduction of DCPIP followed at 600 nm. β-hydroxyacyl-CoA dehydrogenase (β-HAD) activity was determined by incubating homogenate in a buffer containing 100 mM triethanolamine, 451 µM β-nicotinamide adenine dinucleotide and 5 mM EDTA, as previously described [40, 42]. The pyruvate dehydrogenase (PDH) enzyme kinetic were fluorometrically determined using NADH autofluorescence with a spectrofluorometer (FluoroMax Plus-C; Horiba Instruments Inc., Irvine, CA, USA) in a cuvette. For the assay, the 0.2 ml buffer containing 50 mM phosphate buffer containing 10 mM CaCl_2_, 200 mM MgCl_2_, 2 mM rotenone, 8 mM NAD+, 100 mM coenzyme A, and 300 mM thiamine pyrophosphate was used, and the PDH activity was initiated by addition of 10 mM pyruvate. Rates of NADH were converted to picomoles via the NADH standard curve.

### Histological analysis

Terminally in a subset of mice, whole gastrocnemius muscles were extracted, weighed and measured in length; placed in individual molds with optimal cutting temperature medium; frozen in 2-methylbutane cooled by liquid nitrogen; and stored at −80°C. Systematically, 10-µm serial cross-sections were obtained across five standardized regions, 1.5 mm apart, along the entire length of the gastrocnemius muscle (see Figure 7A). The middle region corresponded with the mid-belly of the gastrocnemius muscle and midpoint of the VML injury. The whole muscle was cut proximal to distal. Following the initial ∼100µm into the muscle, 1.5 mm was sectioned, where serial cross-sections of the muscle were obtained. This process was repeated four times for each consecutive region. Across all standardized regions, hematoxylin and eosin staining was performed using standard procedures. NADH staining was performed by incubating muscles at 37°C for 20 minutes in a solution containing 0.2M Tris, 1.5 mM NADH, and 1.5 mM NBT, and succinate dehydrogenase (SDH) staining was performed by incubating muscles at 37°C for 1 hour in a 0.2M sodium phosphate buffer solution, as described previously [24]. At the mid-section only, picrosirius red (Abcam, ab246832) was performed using standard procedures.

Brightfield images were acquired on the TissueScope LE slide scanner (Huron Digital Pathology, St Jacobs, ON, Canada) using a ×20 objective (0.75 NA, 0.5 µm/pixel resolution). Brightfield and polarized images for picrosirius red staining only were acquired using a Nikon Eclipse 200 light microscope (Nikon Instruments Inc., Melville, NY) with diascopic cross-polarized filter using a ×40 objective (E Plan 40x/0.65 OFN20) Whole muscle images were stitched using Nikon Elements D for display purpose only. Additional areas of interest were captured from the defect, border and remaining muscle for analysis only.

Across all five regions of the muscle, quantifications of total fiber number and the proportion of oxidative muscle fibers assessed via mitochondrial co-enzymes SDH and NADH were performed. In the mid-section only, cross-sectional area of individual muscle fibers was measured using the freehand tool. Only fibers with a minimum cross-sectional area of 100 µm^2^ were quantified. All analyses were performed using FIJI [43]. The multipoint tool was used to manually count muscle fibers and the number of darkly (positive) and lightly (negative) stained muscle fibers, indicating high and low metabolic activity, respectively, for SDH and NADH activity, as described previously [24]. Polarized images of picrosirius-stained muscle sections were analyzed in the defect, border and remaining muscle according to the percentage area of green, red, and yellow staining, as previously described [30]. Investigators were blinded during all imaging and analyses.

### Statistical analyses

All statistical analyses were conducted using JMP (version 16.0.0, SAS Institute, Inc.). A Student’s *t*-test was used to compare the means of two groups, such as the direct comparison of peak-isometric torque between uninjured and untreated VML mice. A one-way analysis of variance (ANOVA) was used to compare the means of three or more groups, e.g. when comparing untreated VML to the four experimental groups. A Dunnett *post hoc* test was used to determine which experimental groups were significantly different from untreated VML if the one-way ANOVA *P* values were <0.05. Histological evaluations across five standardized regions of the gastrocnemius muscle were assessed using two-way ANOVA with Tukey’s honestly significant difference (HSD) *post hoc*. Changes in *J*O_2_ and mtMP across ΔG_ATP_ were analyzed by two-way repeated measures ANOVA with one factor being group and the other factor being ΔG_ATP_. Significant interactions were tested with Tukey’s HSD *post hoc* test. Group main effects are reported where significant interactions were not observed. All data were required to pass normality (Shapiro–Wilk) and equal variance tests (Brown-Forsythe F test) before proceeding with the one-way or two-way repeated measures ANOVA. Skeletal muscle fiber cross-sectional area distributions were analyzed by Chi-squared. PEG-DA size distributions did not pass the equal variance ANOVA requirement and were analyzed using the non-parametric Kruskal–Wallis test and therefore are reported as median ± interquartile range. An α level of 0.05 was used for all analyses, and all values are mean ± SD, unless noted in the figure legend.

A power analysis was used to determine sample sizes for the primary study involving physiological outcomes and the secondary study involving histological analysis. For the primary study, a power analysis was completed to detect a minimum 20% improvement in body mass-normalized peak-isometric torque with a typical standard deviation of 70 mN^.^m kg^−1^, which revealed a minimum of seven subjects per cohort. For the secondary study, a power analysis for histology was completed to detect a minimum 10% improvement in total fiber number with a standard deviation of 170 fibers per cross-section, which revealed a minimum of two muscles per group. In both studies, additional mice/muscles were included.

## Results

### VML injury alters muscle size, contractility, stiffness and metabolic function

**Figure 2.**
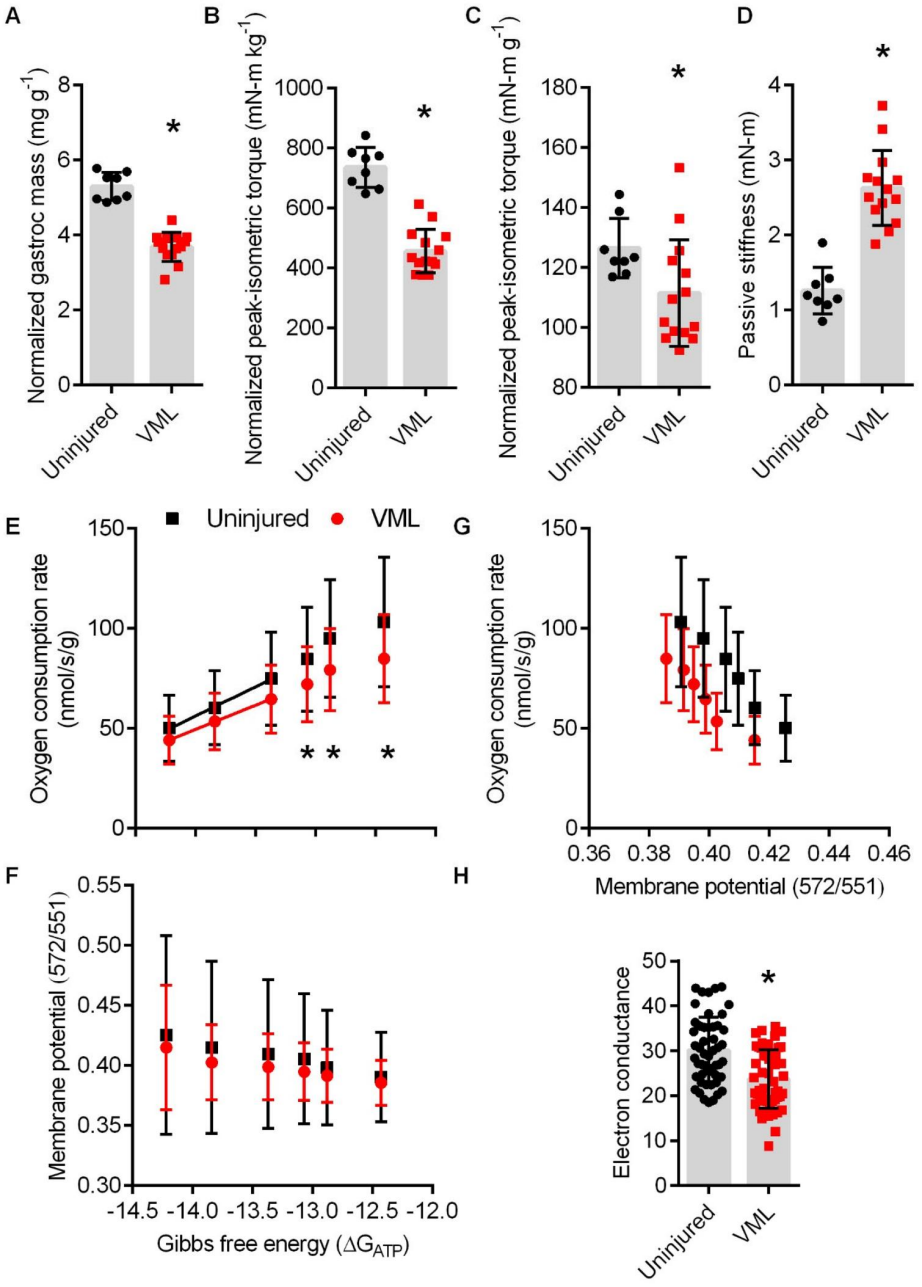
(**A**) Body mass-normalized gastrocnemius muscle mass following volumetric muscle loss (VML) injury (*P* < 0.001). (**B**) Body mass-normalized peak-isometric torque (*P* < 0.001). (**C**) Gastrocnemius muscle mass-normalized peak-isometric torque (*P* = 0.041). (**D**) Passive stiffness about the ankle joint (*P* < 0.001). (**E**) Permeabilized muscle fiber mitochondrial respiration was significantly less at −12.43, −12.88 and −13.07 ΔG_ATP_ (interaction *P* < 0.001; main effect group *P* = 0.005; main effect ΔG_ATP_  *P* < 0.001). (**F**) Mitochondrial membrane potential had greater polarization as ΔG_ATP_ became more negative, independent of group (interaction *P* = 0.068; main effect group *P* = 0.495; main effect ΔG_ATP_  *P* < 0.001). (**G**) Representation of the relationship between membrane potential (*x*-axis) and mitochondrial respiration (*y*-axis) between uninjured and VML-injured. (**H**) Electron conductance (*P* < 0.001). *Significantly different from uninjured. All data are mean±SD. (**A**–**D**) Dots represent individual animals. (**F**) Dots represents individual permeabilized muscle fibers (∼3 fibers per animal).

VML-injured muscle suffers from a permanent loss in muscle mass as well as contractile and metabolic function. Herein, untreated VML mice had 36% less gastrocnemius mass, 38% less peak-isometric torque normalized by body mass, 12% less peak-isometric torque normalized by gastrocnemius muscle mass, and 108% greater passive stiffness about the ankle joint (a physiological marker of fibrotic tissue deposition) compared to uninjured controls (Figure 2A–D). Permeabilized gastrocnemius myofiber bundles were assessed for mitochondrial oxygen consumption (*J*O_2_) over a physiological range of ATP re-synthesis demands using the CK energetic clamp technique previously validated in VML-injured fiber bundles [26]. In agreement with previous acute and chronic VML studies [26, 40], VML injury negatively affected *J*O_2_ across several energetic states (i.e. at −12.43, −12.88 and −13.07 ΔG_ATP_) (Figure 2E). There was no effect of VML injury on mtMP, but the mtMP became more polarized as the energetic states progressed from −12.43 to −14.22 ΔG_ATP_ (Figure 2F). Figure 2G demonstrates the changing relationship between the *J*O_2_ and mtMP during the CK energetic clamp technique. Finally, the linear portion of the *J*O_2_-ΔG_ATP_ was analyzed to determine electron conductance (ease of electron flow through the electron transport chain system), and electron conductance was 21% less in VML compared to uninjured (Figure 2H).

### Characterization of the wheel running

Throughout the remainder of this study, untreated VML-injured mice are compared to four experimental groups: VML-EWR, VML-LWR, VML-Bio, and VML-Bio+F. Both rehabilitation cohorts (VML-EWR, VML-LWR) demonstrated similar voluntary wheel running adherence. After the first initialization week (Week 1), the average daily distance for VML-EWR was 3.4 ± 0.6 km/day (weeks 2–8) and for VML-LWR was 3.4 ± 0.2 km/day (weeks 2–4); these daily distances agree with previous voluntary wheel running studies in VML-injured mice [23, 31]. A progressive intensity treadmill exercise protocol was used to validate that whole-body training adaptations occurred and was conducted at 72 hours prior to euthanasia. Both VML-EWR and VML-LWR completed a significantly greater distance during the exhaustive treadmill test compared to untreated VML controls (*P* ≤ 0.023; VML 1.5 ± 0.6 km, VML-EWR 3.6 ± 1.3 km, and VML-LWR 2.5 ± 0.7 km).

### Biomaterial experimental models

**Figure 3.**
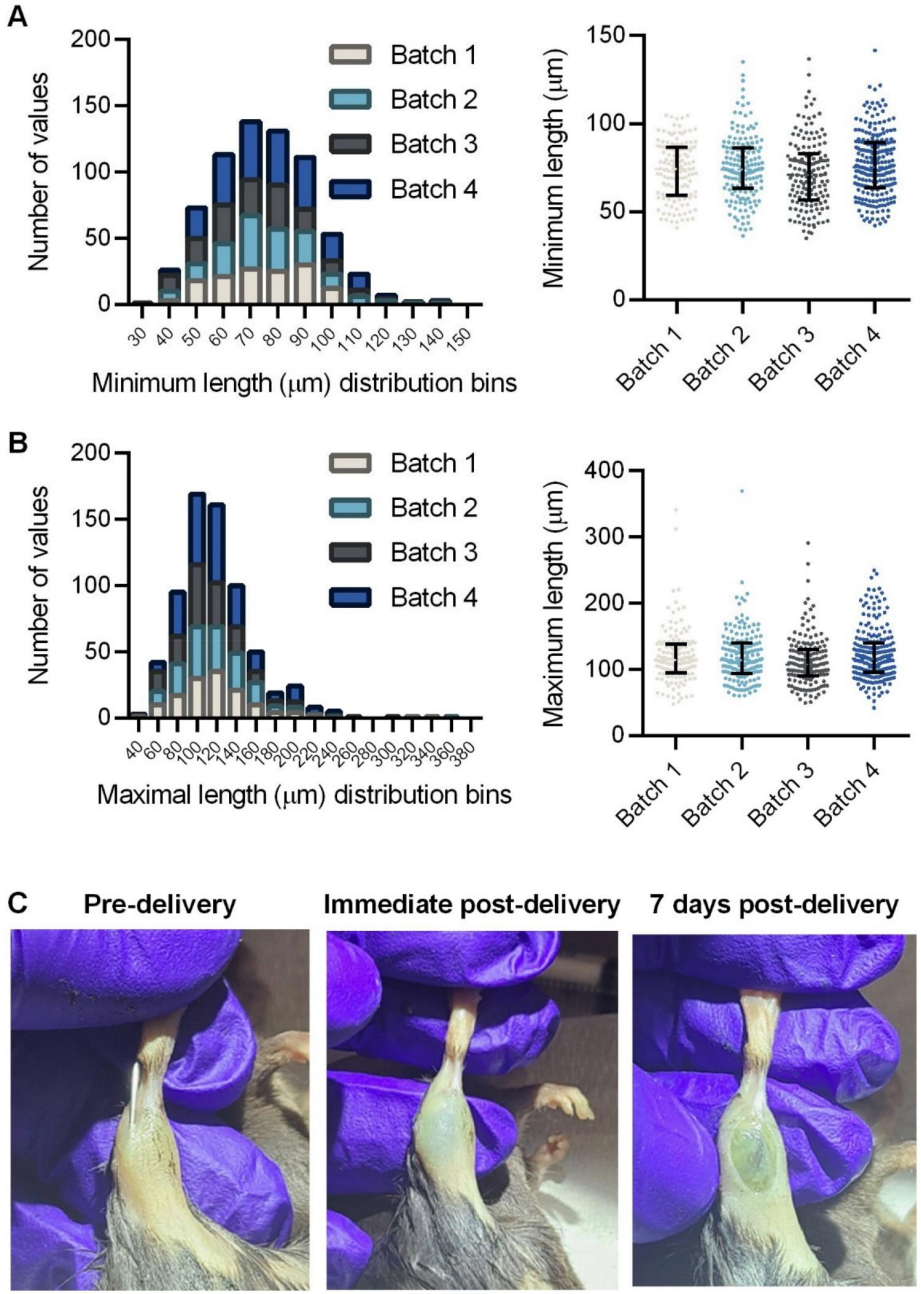
(**A** and **B**) Minimum (*P* = 0.064) and maximum (*P* = 0.099) length distributions for PEG-DA hydrogel fragments from four independent batches were not significantly different. Median and interquartile ranges are shown in the distribution plots (right panels), each dot represents an individual gel fragment. (**C**) Representative anatomical hindlimb images of the animal prior to intramuscular biomaterial delivery and the same animal immediately and 7 days post-delivery. The slight coloration is from EBD-laden biomaterial.

**Figure 4.**
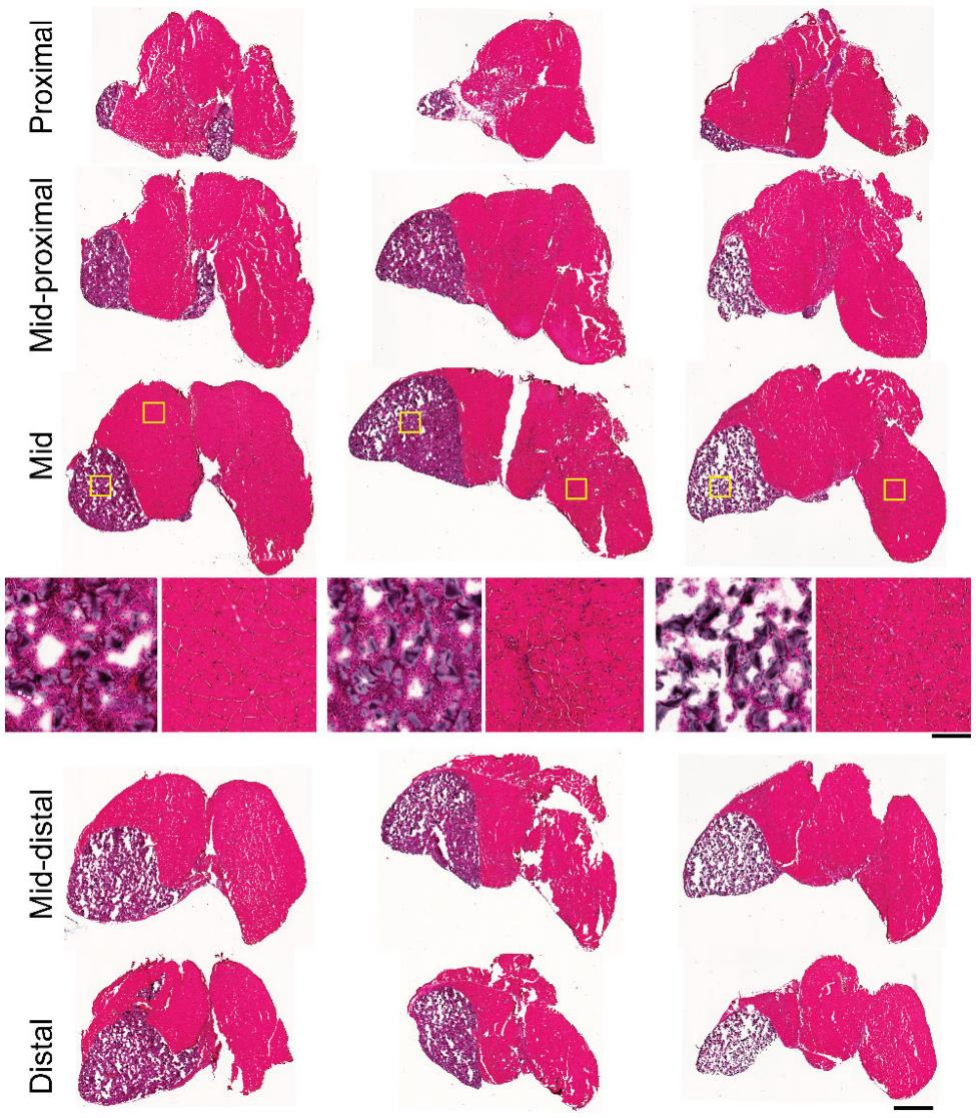
The non-biodegradable PEG-DA was present out to 2 months post-delivery. Representative hematoxylin and eosin-stained images are from the gastrocnemius muscle of three animals at 2 months post-biomaterial delivery. The biomaterial can be observed in the lower-left quadrant, or the medial head, of each gastrocnemius muscle. Regions of interest are marked within the mid-belly and shown at a higher magnification immediately below the mid-belly images, taken from the biomaterial region and the remaining muscle region. The scale bar is 125 µm. There is evidence of biomaterial presence along the full length of the muscle, proximal to distal. Scale bar for full muscle images is 1 mm.

Biomaterial batch-to-batch variability was determined by analyzing fragment size distribution for four independent batches using both the minimum and maximal lengths (Figure 3A and B). There were no significant differences in minimum or maximum length distribution across batches, and for all batches the median minimum length was 74 µm and the median maximum length was 114 µm. Evans blue dye was conjugated to the biomaterial prior to intramuscular injection to verify stability within the tissue. Visually, the dye was noticeable immediately after injection and at 7 days post-injection (Figure 3C). Furthermore, as the biomaterial is not biodegradable, there was histological evidence of its presence within the muscle at 30 days post-injection (Figure 4).

**Figure 5.**
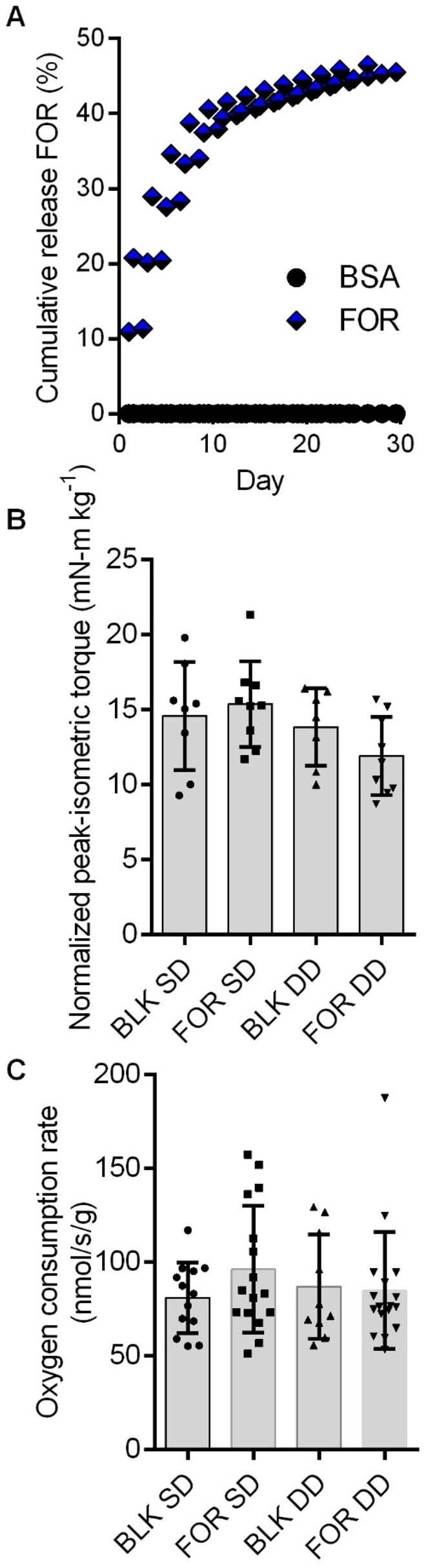
(**A**) *In vitro* formoterol (for) release kinetics from PEG-DA biomaterial for three independent experiments shown in contrast to cumulative formoterol release for bovine serum albumin (BSA)-laden PEG-DA (control). Cumulative formoterol release was not different among the three formoterol-laden biomaterials (*P* = 0.979) but was significantly greater than BSA-laden biomaterials (*P* < 0.001). (**B**) Body mass-normalized peak-isometric torque at 1 month post-injury was not different for groups that received a single blank biomaterial (BLK SD), and double-dose blank biomaterial (BLK DD), a single-dose formoterol-laded biomaterial (for SD), or a double-dose formoterol-laden biomaterial (for DD) (*P* = 0.053). Individual data points represent a single mouse. (**C**) There were no statistically significant differences in permeabilized muscle fiber mitochondrial *J*O_2_ across groups (*P* = 0.746). Individual data points represent an individual permeabilized muscle fiber bundle (2–3 per animal). Data are mean±SD.

The Kd values from three independent biomaterial release assays were 4.2, 4.0 and 3.8 days and the Bmax values were 53%, 54% and 52% cumulative release over 30 days (Figure 5A). This indicates a strong release curve within the first week and a slow plateau out to 1 month. Therefore, we decided to test the effects of formoterol-laden biomaterial at 1 month post-delivery.

Next, we conducted feasibility studies to determine at what time post-VML injury to deliver the formoterol-laden biomaterial. We examined peak-isometric torque and permeabilized muscle fiber mitochondrial *J*O_2_ after administering formoterol-laden biomaterial with a single dose (i.e. 3-days post-VML) and a double dose (i.e. 3 and 14 days post-VML) compared to biomaterial alone. At 1 month post-VML, there was a trend in the peak-isometric torque data for single-dose formoterol being greater than single-dose blank biomaterial, but there was no statistical difference among groups for *J*O_2_ (Figure 5B and C).

Moving forward we decided to deliver a single dose at 1 month post-injury. This decision was made based on the single-dose vs double-dose feasibility data and the extensive literature of inflammation after VML injury that extends out to 1 month [20, 44, 45]. Specifically, we considered the possibility that our feasibility studies failed to improve VML-injured muscle function because we introduced a foreign agent (i.e. biomaterial) during a time of heightened inflammation.

### Differential adaptations in muscle mass and contractility with regenerative rehabilitation and PEG biomaterial

**Figure 6.**
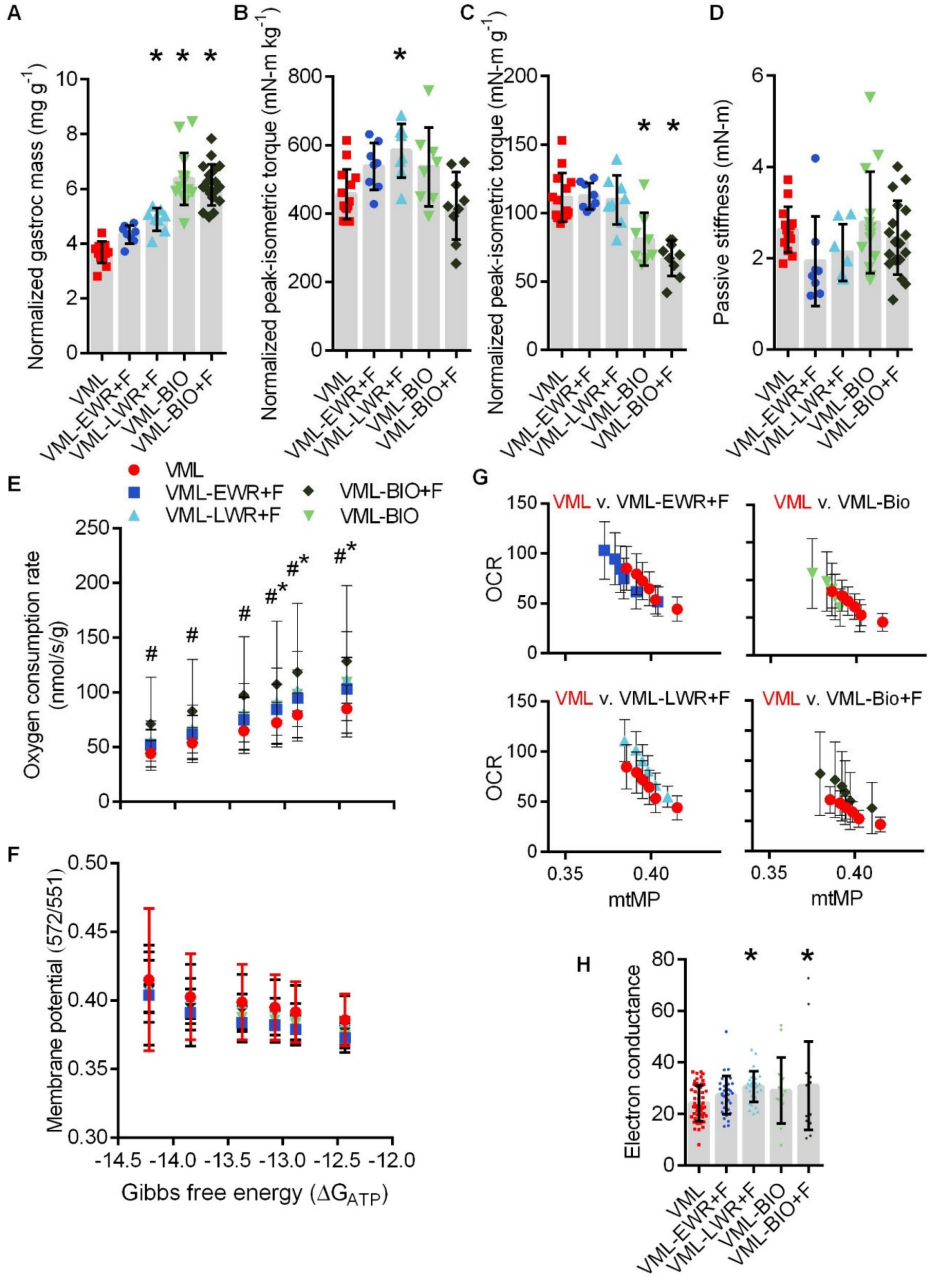
(**A**) Body mass-normalized gastrocnemius masses (*P* < 0.001), *Difference from untreated volumetric muscle loss (VML). (**B**) Body mass-normalized peak-isometric torque (*P* = 0.002), *difference from untreated VML. (**C**) Muscle mass-normalized peak-isometric torque (*P* < 0.001), *Difference from untreated VML. (**D**) Passive stiffness about the ankle joint (*P* = 0.161). (**E**) *J*O_2_ across clamped ΔG_ATP_ states (interaction *P* < 0.001; main effect ΔG_ATP_  *P* < 0.001; main effect group *P* < 0.001), #VML-Bio+F greater than untreated VML, *VML-LWR greater than untreated VML. (**F**) Mitochondrial membrane potential across ΔG_ATP_ (interaction *P* = 0.977, main effect ΔG_ATP_  *P* < 0.001; main effect group *P* = 0.073). (**G**) *J*O_2_ (*y*-axis) plotted against mitochondrial membrane potential (*x*-axis) representing the six clamped ΔG_ATP_ stages for each experimental group compared to untreated VML. Shifts ‘up’ represent a greater *J*O_2_ and shift ‘left’ represent hypo-polarized mtMP. (**H**) Electron conductance (*P* = 0.012), *Difference from untreated VML. All data are mean±SD. (**A**–**D**) Dots represent individual animals. (**F**) Dots represent individual permeabilized muscle fibers (∼3 per animal).

At 2 months post-VML, no experimental VML groups had a terminal body mass different than VML untreated; however, across experimental VML groups there was variability in terminal body mass; therefore, gastrocnemius muscle mass and peak-isometric torque were normalized to body mass (Figure 6A and B). Normalized muscle mass was greater in mice receiving the biomaterial and in the VML-LWR mice compared to VML untreated. Body mass-normalized peak-isometric torque was 28% greater in VML-LWR mice compared to VML untreated (Figure 6B). There was no difference between VML-LWR and VML untreated when peak-isometric torque was normalized to muscle mass, an indicator of muscle quality (Figure 6C), suggesting that strength gains in the VML-LWR mice were mostly driven by larger muscle masses but not an improved muscle quality. Both biomaterial groups had significantly lower muscle mass-normalized torques (≥-27%), suggesting a worsened muscle quality. *In vivo* passive stiffness was not altered by any experimental condition in comparison to VML untreated (Figure 6D).

### Favorable adaptations in mitochondrial bioenergetics with regenerative rehabilitation and formoterol-laden biomaterial

Permeabilized muscle fibers *J*O_2_ and mtMP were simultaneously assessed during the CK clamp technique. Independent of group, *J*O_2_ decreased with sequential PCr steps in agreement with previous studies that VML-injured fibers remain sensitive to the CK clamp technique [26, 40]. There was a significant interaction between *J*O_2_ and ΔG_ATP_ (*P* < 0.001). At ΔG_ATP_ −12.43, −12.88, −13.07 and −13.37, both VML-LWR and VML-Bio+F had greater *J*O_2_ compared to untreated VML, and VML-Bio+F was greater than VML at −13.84 and −14.22 ΔG_ATP_ (Figure 6E).

There was only a significant effect of ΔG_ATP_ for mtMP as membranes became more polarized with decreasing energetic demands (Figure 6F); however, there was a trend for experimental groups being significantly different compared to VML, independent of ΔG_ATP_ (*P* = 0.07). This trend can be better visualized when contrasting *J*O_2_-MMP plots for VML vs. experimental groups (Figure 6G). mtMP reflects the proton motive force across the inner-mitochondrial membrane and, in part, influences the ability of Complex V (ATP synthase) to displace the ATP:ADP ratio from equilibrium during changing energetic demands. These plots suggest that the mitochondria in the untreated VML cohort were less able to maintain bioenergetic stability compared to the experimental groups. Finally, electron conductance was ∼27% greater in VML-LWR and VML-Bio+F compared to untreated VML (*P* = 0.014, Figure 6H).

Due to the changes in *J*O_2_ and electron conductance, the activities of individual mitochondrial enzymes that can influence metabolism were assessed (Table 1). PDH catalyzes the glycolysis end-product pyruvate into acetyl-CoA for Krebs cycle entry and β-HAD catalyzes the third step of β-oxidation that has a common end-product to PDH, acetyl-CoA. There were no significant differences between VML untreated and any experimental group for these two mitochondrial enzymes. CS catalyzes the first step of the Krebs cycle by converting acetyl-CoA (from PDH or β-oxidation) into citrate. VML-Bio+F had less CS activity compared to untreated VML. Electron conductance starts with the oxidation of reduced metabolic equivalents (e.g. NADH) at complex I and complex II of the ETS. Complex I enzyme activity was significantly greater in VML-EWR compared to VML and Complex II enzyme activity was significantly greater in VML-Bio+F compared to VML.

**Table 1.** Mitochondrial enzyme activities

		Experimental group					
Enzymatic activity	Uninjured	VML	VML-EWR+F	VML-LWR+F	VML-Bio	VML-Bio+F	OWA *P*-value
Citrate synthase (µmol/min/g)	349.8 ± 126.9^a, b^	369.4 ± 90.9^a, b^	366.7 ± 70.4^a, b^	465.1 ± 127.0^a^	270 ± 88.4^b^	220.6 ± 51.76^b^	0.002
Complex I (µmol/min/g)	18.3 ± 5.0	18.3 ± 5.0	26.3 ± 3.5^#^	15.7 ± 5.2	19.3 ± 7	21.7 ± 1.9	0.022
Complex II (µmol/min/g)	16.7 ± 10.4	16.7 ± 10.4	21.5 ± 10.0	13.69 ± 9.0	24.1 ± 6.3	23.8 ± 7.0	0.057
β-HAD (µmol/min/g)	42.3 ± 9.0	42.3 ± 9.0	37.0 ± 11.4	50.0 ± 13.8	33.0 ± 8.9	46.0 ± 8.2	0.005
PDH (pmol/s/mg)	263.4 ± 169.4	263.4 ± 169.4	224.0 ± 98.7	200.7 ± 50.8	145.9 ± 95.4	152 ± 84.1	0.154

OWA, one-way ANOVA.

### Histological examination

A rigorous histological examination of the gastrocnemius muscle was completed on additional cohorts of (i) uninjured, (ii) untreated VML, (iii) VML oral formoterol-only, (iv) VML-LWR+F and (v) VML-Bio+F mice in order to learn more about the morphological changes associated with formoterol that may contribute to the improved physiological function. Only VML-LWR+F and VML-Bio+F groups were included based on the most promising physiological findings reported above.

**Figure 7.**
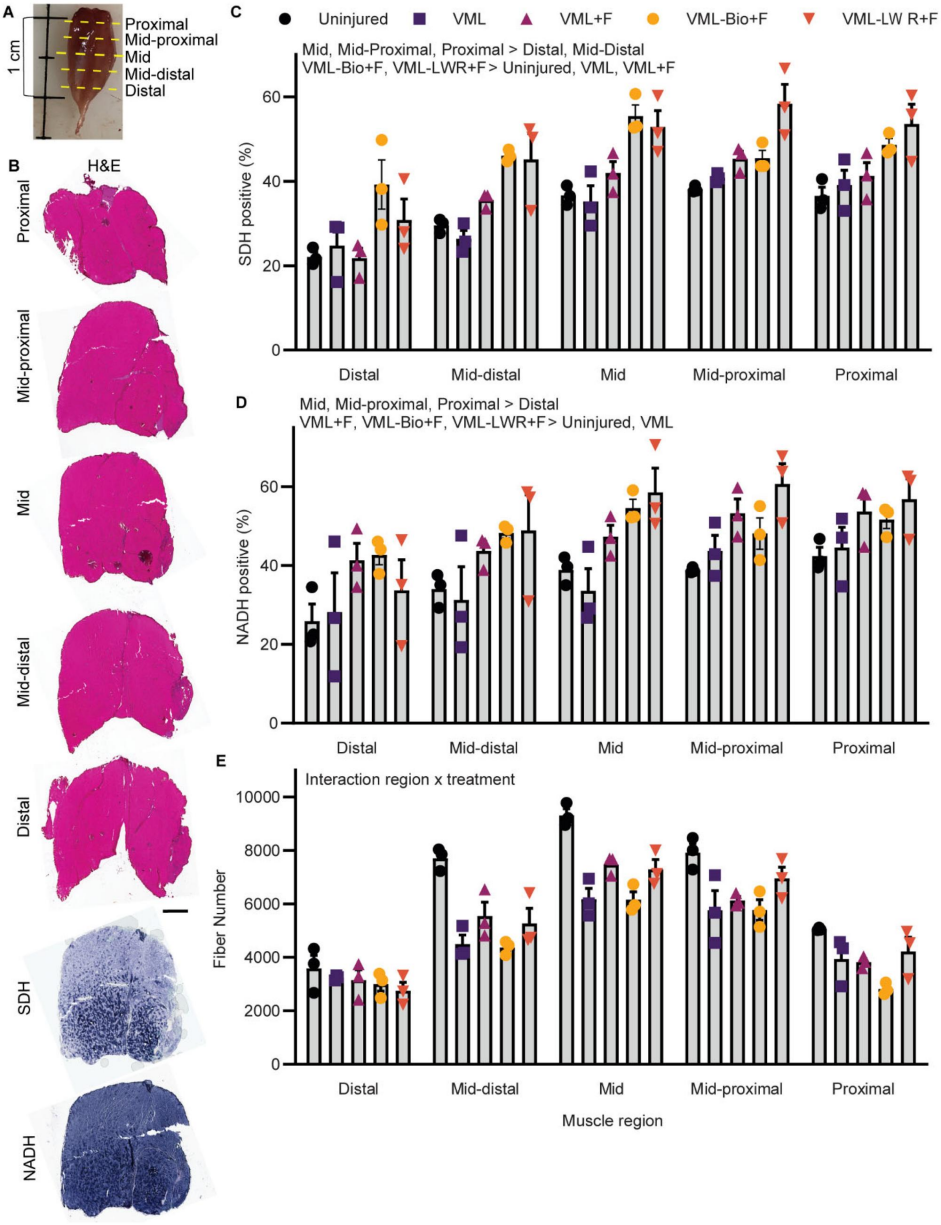
(**A**) Representative gastrocnemius muscle showing location of distal to proximal muscle sections. (**B**) Representative histological sections from a VML-injured muscle; scale bar 1 mm. (**C**) Percent positive SDH fibers at each region (interaction *P* = 0.170; main effect of group *P* < 0.001; main effect of region *P* < 0.001). (**D**) Percent positive NADH fibers at each region (interaction *P* = 0.838; main effect of group *P* < 0.001; main effect of region *P* < 0.001). Representative stained images for SDH and NADH from the mid-belly shown, notably this is the same muscle as in (**B**). (**E**) Total fiber numbers at each region (interaction *P* = 0.020; main effect of group *P* < 0.001; main effect of region *P* < 0.001). All data are mean±SD. Symbols represent individual animals.

For total fiber number and markers of oxidative capacity (SDH^+^ and NADH^+^ staining), serial sections were cut through the muscle starting at the proximal end (proximal and mid-proximal) to the mid-belly (mid) and finishing at the distal end (mid-distal and distal) (Figure 7A and B). There was no significant interaction between region and experimental group for SDH^+^ fibers; however, there was a main effect of experimental group, independent of histological region. Both VML-LWR+F and VML-Bio+F had a greater percentage of SDH^+^ fibers compared to uninjured and untreated VML (Figure 7C). There was no significant interaction between region and experimental group for NADH^+^ fibers; however, there was a main effect of experimental group, independent of histological zone. All three formoterol groups (VML-LWR+F, VML-Bio+F and VML oral formoterol-only) had a greater percentage of NADH^+^ fibers compared to uninjured and untreated VML (Figure 7D).

For total fiber number, there was a significant interaction between histological region and experimental group (Figure 7E). At the most distal histological region, there were no statistical differences across groups. At the mid and mid-distal histological regions, all VML-injured experimental groups had fewer total fibers compared to uninjured. At the mid-proximal histological region, all VML-injured experimental groups except VML-LWR+F had significantly fewer total fibers compared to control. At the proximal histological region, only VML-Bio+F had significantly fewer total fibers compared to uninjured.

Collagen packing was visualized by staining for picrosirius red. Cross-polarized filters display a spectrum of red-to-green hues by exploiting the birefringent properties of collagen and eliminate non-collagenous structures. Because collagen I (thicker) stains red/orange/yellow and collagen III (thinner) stains green, these hue differences were previously thought to distinguish collagen I from collagen III [46]. This, however, is unlikely given that immature collagen I may be relatively thin. Hue differences instead are more likely indicative of collagen packing, where densely packed collagen appears red/orange/yellow and loosely packed collagen appears green. Therefore, the percentage area of collagen stained red/orange/yellow and the percentage area stained green were quantified in the gastrocnemius muscles. Mid-belly muscle sections were evaluated at three systematically selected areas of interest: the defect area, the remaining muscle, and the border between the defect and remaining muscle.

**Figure 8.**
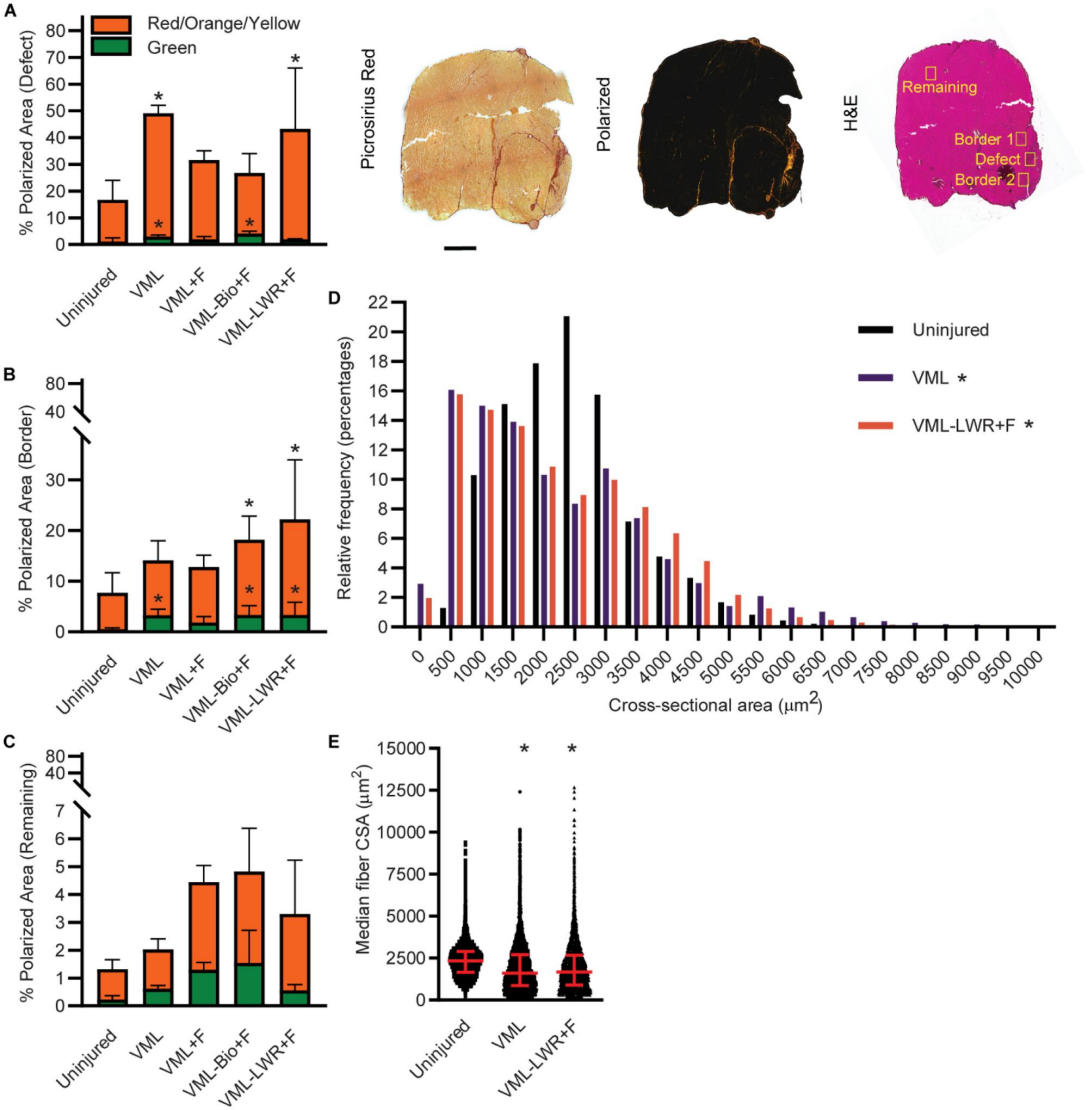
Within the mid-section of the muscle only, representative stained images for picrosirius red, polarized view, and H&E are shown, notably this is the same muscle as in Figure 7B. Defined VML regions of interest (defect, border 1, border 2, and remaining) are shown in the H&E representation. (**A**) VML defect area analysis of percent muscle area that is densely packed (red/orange/yellow) (*P* = 0.013) and loosely packed (green) (*P* < 0.001) collagen. *Statistically different than uninjured. (**B**) VML border area analysis of densely packed (*P* = 0.007) and loosely packed (*P* = 0.011) collagen. *Statistically different than uninjured. (**C**) VML remaining muscle area analysis of densely packed (*P* = 0.127) and loosely packed (*P* = 0.076) collagen. (**D**) Relative frequency of fiber cross-sectional area (*P* < 0.001). Significant distributions shifts are noted for VML and VML-LWR+F relative to uninjured. (**E**) Median fiber cross-sectional area (*P* < 0.001). Data are median and interquartile range. *Significantly different that uninjured.

At the defect (Figure 8A), all VML-injured groups had significantly greater green polarized percent area (loosely packed collagen) compared to uninjured. Both untreated VML and VML-LWR+F had greater red/orange/yellow percent of polarized area (densely packed collagen) compared to uninjured. At the border (Figure 8B), all VML-injured groups with the exception of oral formoterol-only had significantly greater green percent of polarized area compared to uninjured. Both VML-Bio+F and VML-LWR+F had greater red/orange/yellow percent of polarized area compared to uninjured at the border. There was no significant difference across groups within the remaining muscle (Figure 8C). The ratio of loosely packed to densely packed collagen (red/orange/yellow-to-green ratio) was not significantly different across VML-injured groups (*P* ≥ 0.469).

All individual fiber cross-sectional areas were determined at the mid-belly only, for uninjured, VML, and VML-LWR+F. The mid-belly was selected because it represents the initial VML injury and the maximal thickness point of the muscle (i.e. the region with the most total fibers). Only the VML-LWR+F experimental group was selected based on its moderately promising total fiber number results. There was a significant left distribution shift in fiber cross-sectional area for VML and VML-LWR+F compared to uninjured controls (Figure 8D), indicating a relatively greater population of small cross-sectional area fibers. The median cross-sectional area was also greater in uninjured compared to both VML and VML-LWR+F (Figure 8E).

## Discussion

This study was designed to test if a regenerative rehabilitation paradigm or biomaterial delivery of formoterol could improve upon the previously published benefits of formoterol in the context of VML injury [11, 28, 29]. There are three primary conclusions based upon the results. First, there is modest evidence to support that this study’s iteration of regenerative rehabilitation improved upon the established effectiveness of formoterol. Second, the non-biodegradable biomaterial negatively affected muscle contractile quality, yet was effective in promoting greater mitochondrial function. Third, this work advances and strengthens the therapeutic modality of formoterol for improving muscle function after a VML injury. Each conclusion will be expanded upon in the following sections.

### Moderate evidence of enhanced formoterol effect with regenerative rehabilitation

Previous studies investigating the therapeutic potential of formoterol in the context of VML injury used an oral delivery approach without any rehabilitation intervention. There is moderate evidence that regenerative rehabilitation in this study outperformed (indirectly through comparison of published results) the previous formoterol delivery without rehabilitation. For example, the 28% greater body mass-normalized peak-isometric torque with formoterol combined with late-wheel running (VML-LWR+F) is higher than the range reported with formoterol treatment alone in previous studies (+14–18%, [11, 28]). Similarly, body mass-normalized gastrocnemius muscle mass was 32% greater in VML-LWR+F mice compared to untreated VML. This percent improvement is comparatively greater than the published range for formoterol alone (+7–20%, [11, 28]). We directly compared regenerative rehabilitation (VML-LWR+F) to oral formoterol-only for total fiber numbers across five regions of the gastrocnemius muscle, and there was a ∼4.5% greater number of total fibers at the mid-proximal region (i.e. just superior to the VML defect) of muscles from VML-LWR+F. These modest findings are encouraging for regenerative rehabilitation involving formoterol.

The qualitative comparison of gastrocnemius muscle mass and muscle strength improvement with regenerative rehabilitation relative to formoterol alone is optimistic but should be considered with caution. First, this study lacked a direct comparison of physiological responses between formoterol-only and the regenerative rehabilitation. Second, regenerative rehabilitation did not yield a relative improvement in ‘muscle quality’, as indicated by muscle mass-normalized torque. The direct histological comparison of collagen content in the VML-LWR+F and VML formoterol-only groups provides greater insight into this critique. Collagen is denser than muscle (1.3 vs 1.06 g/cm^3^, respectively), and muscle from formoterol-only mice had similar loosely and densely packed collagen at the defect and border locations of the VML injury compared to uninjured muscle, whereas the regenerative rehabilitation group, VML-LWR+F, had greater. This suggests that collagen content is contributing in part to the relative muscle mass difference between regenerative rehabilitation and formoterol alone and potentially influencing the quality of the muscle. Lastly, the ∼30% greater *J*O_2_ with formoterol plus wheel running (VML-LWR+F) agrees with, but is not better than, the 36% improvement in permeabilized muscle fiber mitochondrial respiration reported using formoterol alone [11]. Taken together, formoterol and delayed rehabilitation is a promising first step toward developing an effective regenerative rehabilitation approach for VML, yet more optimization is needed, and the combined approach remains far from curative.

### Non-biodegradable PEG, a mixed initial step toward direct formoterol delivery

Non-biodegradable and biodegradable biomaterials are commonly used throughout various stages of regenerative medicine development. While each material has strengths and weaknesses, our laboratory made the choice to first try a non-biodegradable biomaterial for the delivery of formoterol. The non-biodegradable PEG-DA used here unquestionably had a negative influence on muscle contractile function and morphology (i.e. less muscle strength, fewer total fiber numbers), but did yield some promising outcomes to warrant expansion in future experiments, e.g. greater metabolic function.

Direct delivery of formoterol to the injured muscle is an important objective for regenerative medicine to reduce the overall drug amount required for each subject (and to reduce costs) and because prolonged β_2_-adrenergic receptor agonists may have systemic effects [47] (although there is evidence to the contrary as well [48]), that may be unwanted in the context of VML. One obstacle to direct muscle delivery is the therapeutic window of the dose. For example, directly injected into the hindlimbs of rats, formoterol promotes a significant increase in muscle mass at 5 days post-injection (100 µg dose) [49]. When this single dose of formoterol was intramuscularly delivered to animals following myotoxin-induced muscle injury, there was greater recovery of muscle mass at 7 days post-injury; however, the therapeutic window appeared to be brief, as formoterol-treated animals did not demonstrate greater muscle masses compared to injured control rats at 10 and 14 days post-injury [49]. The therapeutic window was extended when formoterol was delivered daily via intraperitoneal injections, resulting in greater muscle mass and function in a mouse model of Duchenne muscular dystrophy [50] and in aging rats [51]. Translationally, daily intraperitoneal injections are considered hurdles due to potential systemic effects and patient adherence [52, 53]. Our pursuit of direct formoterol delivery by biomaterial is that the biomaterial could potentially be delivered monthly and provide a longer therapeutic window directly to the injured muscle. The *in vitro* release kinetics and muscle metabolic function reported here provide motivation to optimize a biodegradable option in future studies. That said, there are other potential delivery options available to pursue outside of a biomaterial such as a transdermal delivery model that has been used for nicotine [54], lidocaine [55] and tulobuterol (an alternative β-adrenergic receptor agonists [56]).

### Building evidence of formoterol effectiveness in VML

Formoterol and other β-adrenergic receptor agonists have shown broad benefit across several skeletal muscle conditions, and this work strengthens the efficacy of β-adrenergic receptor agonism for VML. β-adrenergic receptor agonists are associated with greater recovery of muscle force following myotoxic muscle injury in rodents [57], and an increase in voluntary muscle strength in both able-bodied, healthy men [8] and patients with a spinal cord injury [58]. β-agonist-induced skeletal muscle adaptations are due in part to stimulating greater protein synthesis by activating cyclic AMP-PKA and downstream activation of Akt [7], a molecular regulator of protein turnover balance in skeletal muscle.

One might predict that the remaining muscle after VML injury, tasked with maintaining functional capacity with a smaller mass, will display similar activation of protein synthesis pathways (e.g. Akt) given that surgical synergistic ablation of the gastrocnemius muscle causes such adaptations in the overloaded plantaris muscle [59, 60]. However, there is currently minimal evidence of any such molecular adaptation in VML-injured muscle [28, 31], and even with β-adrenergic receptor agonist treatment, there is no notable change in protein expression patterns for markers of muscle atrophy (e.g. MuRF1) and protein synthesis (e.g. Akt) [28]. The lack of robust molecular signaling changes is supported by the inability of regenerative rehabilitation consisting of formoterol and wheel running to result in any changes in fiber cross-sectional area.

β-Adrenergic receptor agonist treatment is known to influence muscle fiber-type and to have greater potency in slow-twitch (‘oxidative’) fibers that have greater density of β-adrenergic receptors [61, 62]. We and others have shown that β-agonism can stimulate maximal *J*O_2_ [11, 63, 64] and influence whole-body energy metabolism [28, 63]. Herein, we also show that β-agonism affected electron conductance across the ETS, and this is consistent with another report of β-agonism enhancing the ETS by way of enhanced proton conductance [65]. In total, the histological (NADH/SDH) and physiological (*J*O_2_) metabolic changes associated with formoterol (either regenerative rehabilitation or biomaterial) are much more robust and consistent than muscle size and strength changes in the context of VML. It is unclear why the metabolic effects are more consistent. At least one study has demonstrated that β-blocker inhibition attenuates β-agonism-induced changes in *J*O_2_ [64], suggesting a critical role for the presence of the receptor, while we have previously reported that VML injury is associated with a decrease in β-receptor presence that was not reversed in the presence of formoterol alone. The extent to which rehabilitation in addition to oral formoterol alone and/or direct delivery of formoterol via a biomaterial influenced β-receptor presence in VML injury muscle was not explored, but this presents one future direction to better understanding formoterol efficacy in VML.

In summary, our findings advance the efficacy of formoterol as an adjuvant therapeutic for skeletal muscle function after VML injury. In the context of VML, evidence supports formoterol having a greater effect on metabolic compared to contractile function, yet the direct mechanism(s) remain unknown. Regenerative rehabilitation and regenerative medicine approaches using formoterol in this study produced promising results that were far from curative and require greater optimization. For regenerative rehabilitation, delayed rehabilitation was more effective than early rehabilitation, and the influence of rehabilitation on formoterol signaling through β-adrenergic receptors needs to be explored. For regenerative medicine, a biodegradable biomaterial needs to be developed to test if the benefits of direct muscle delivery of formoterol can be recapitulated without the negative consequences of the non-biodegradable biomaterial taking up permanent residence within the remaining muscle.

## Data Availability

The datasets used and/or analyzed during the current study are primarily presented in the current manuscript and are available from the corresponding author on reasonable request.
